# FTO-mediated SMAD2 m6A modification protects cartilage against Osteoarthritis

**DOI:** 10.1038/s12276-024-01330-y

**Published:** 2024-10-03

**Authors:** Hongyi Zhou, Ziang Xie, Yu Qian, Weiyu Ni, Lei Cui, Xiangqian Fang, Shuanglin Wan, Xiangde Zhao, An Qin, Shunwu Fan, Yizheng Wu

**Affiliations:** 1 Department of Orthopaedic Surgery, Sir Run Run Shaw Hospital, Zhejiang University School of Medicine, Hangzhou, China; 2 Key Laboratory of Musculoskeletal System Degeneration and Regeneration Translational Research of Zhejiang Province, Hangzhou, China; 3 Department of Orthopaedics and Traumatology, The First Affiliated Hospital of Zhejiang Chinese Medical University (Zhejiang Provincial Hospital of Chinese Medicine), Hangzhou, China; 4 Collaborative Innovation Centre of Regenerative Medicine and Medical BioResource Development and Application Co-constructed by the Province and Ministry, Guangxi Medical University, Nanning, China; 5 Department of Orthopaedics, Shanghai Key Laboratory of Orthopaedic Implants, Shanghai Ninth People’s Hospital, Shanghai Jiaotong University School of Medicine, Shanghai, China

**Keywords:** Cartilage development, Predictive markers

## Abstract

N6-methyladenosine (m6A) modification is one of the most prevalent forms of epigenetic modification and plays an important role in the development of degenerative diseases such as osteoarthritis (OA). However, the evidence concerning the role of m6A modification in OA is insufficient. Here, m6A modification was increased in human OA cartilage and degenerated chondrocytes. Among all of the m6A enzymes, the expression of the demethylase fat mass and obesity-associated protein (FTO) decreased dramatically. Conditional knockout of FTO in chondrocytes accelerates OA progression. FTO transcription is regulated by runt-related transcription factor-1 (RUNX1). Reduced FTO elevates m6A modification at the adenosine N6 position in SMAD family member 2 (SMAD2) mRNA, whose stability is subsequently modulated by the recruited m6A reader protein YTH N6-methyladenosine RNA binding protein F2 (YTHDF2). Collectively, these findings reveal the function and mechanism of the m6A family member FTO in OA progression. Therefore, reducing m6A modification to increase SMAD2 stability by activating FTO might be a potential therapeutic strategy for OA treatment.

## Epigenetic insights: FTO’s role in osteoarthritis progression

Osteoarthritis is a widespread joint disease-causing pain and disability. It involves the deterioration of joint cartilage and bone, but the exact reasons are unclear. This study aimed to investigate the role of a specific change in RNA molecules, called N6-methyladenosine, in OA development. The researchers focused on the enzyme FTO, which can remove this change, and its effect on cartilage cells in mice. They used different methods, including genetic modification to create mice lacking FTO in their cartilage cells, to see how changes in m6A levels affect OA progression. The main findings show that reducing FTO expression worsens OA progression by affecting the stability and function of specific RNA molecules in cartilage cells. The researchers conclude that targeting the m6A change pathway, especially by modulating FTO activity, could provide new treatment strategies for OA.

This summary was initially drafted using artificial intelligence, then revised and fact-checked by the author.

## Introduction

Osteoarthritis (OA) is the most prevalent degenerative joint disease, causing a significant socioeconomic burden worldwide^1^. It has been linked to aging, obesity, and acute injury. Articular cartilage destruction, synovial hyperplasia, osteophyte formation, and subchondral bone remodeling are the primary features of OA^2,3^. Dysregulation of anabolic and catabolic pathways in chondrocytes leads to extracellular matrix (ECM) degeneration^4,5^.

Many studies have revealed important molecular mechanisms that regulate OA development. Several intracellular proteins play essential roles in OA progression. SOX9 levels in OA cartilage decrease^6^, and Runt-related transcription factor-1 (Runx1) slows OA progression^7^. On the other hand, extracellular proteins are crucial in OA. The expression of MMP13 and cathepsin K is increased in OA^8^. In addition, interleukin-1β (IL-1β) and tumor necrosis factor α (TNF-α) are important OA mediators^5^. Moreover, the expression of transforming growth factor β (TGFβ) and SMAD family member 2 (SMAD2) is decreased in OA^9^. Moreover, different modifications are required for OA regulation. For example, asymmetric dimethylarginine inhibited the deubiquitination effect of USP7 on SOX9 and exacerbated OA progression^10^. Kindlin-2 deficiency inhibited SMAD2 phosphorylation and chondrocyte differentiation^11^. However, the regulation of OA by various modifications requires further investigation.

N6-methyladenosine (m6A) modification is one of the most common internal modifications of eukaryotic mRNAs and usually occurs within the DRACH consensus motif (D = A, G, or U; R = A or G; H = A, U, or C)^12–14^. Research has reported that m6A modification can be regulated by three types of proteins, namely, “writers,” “erasers,” and “readers.” Two common “writers,” methyltransferase-like 3 and 14 (METTL3 and METTL14), are important components of the m6A methyltransferase complex (MTC)^15,16^. Another important “writer” is Wilms’ tumor 1-associating protein (WTAP), which may promote m6A installation and recruit METTL3 and METTL14 into nuclear speckles^17^. Moreover, m6A can be reversed simultaneously by at least two specific biomarkers, fat mass and obesity-associated protein (FTO) and AlkB homolog 5 (ALKBH5), which convert m6A to adenosine (A)^18,19^. This modification recruits diverse m6A-binding proteins, including YTH family members, a class of m6A “readers” that includes YTHDF1/2/3. YTHDF1 promotes mRNA translation, YTHDF2 promotes mRNA degradation, and YTHDF3 plays a synergistic role^20–22^. Collectively, these studies highlight that m6A modification regulates RNA metabolism, such as stability and translation, and is linked to the occurrence and progression of various human diseases.

Several studies have investigated the relationship between m6A and OA. Among them, the function of METTL3 has been extensively investigated. For example, METTL3 regulates the pyroptosis-related protein NLRP3 to alleviate OA^23^. By regulating METTL3, the autophagy-related protein ATG7 regulates senescence and OA progression^24^. TIMPs and MMPs are associated with METTL3, which influences extracellular matrix (ECM) degradation during OA progression^25^. Similarly, to regulate ECM synthesis in chondrocytes, the NF-κB signaling pathway is linked to METTL3^26^. Nevertheless, the functions of other m6A family members in OA remain unclear.

FTO was one of the earliest “erasers” identified^18^. To alleviate OA, FTO overexpression regulates miR-515-5p^27^. In addition, FTO-mediated m6A modifications regulate AC008440.5 transcription and slow OA progression^28^. RUNX1 is a transcription factor that plays an important role in OA^29^. Previous studies have revealed that the overexpression of RUNX1 can help alleviate OA^7^. As an important protein in the TGFβ signaling pathway, SMAD2 can alleviate the progression of OA by regulating FBXO6^30^. In this study, we revealed that m6A is related to OA and that FTO may play a predominant role in OA progression. To investigate the role of FTO in OA, its upstream transcription factor RUNX1 and how FTO regulates its downstream target gene SMAD2, we generated chondrocyte-specific FTO-knockout (FTO^fl/fl^, Col2a1-CreER^T2^) mice. FTO was found to be closely associated with human OA progression, whereas FTO knockout downregulated SMAD2 and promoted OA progression in a mouse model. Therefore, FTO is a potential molecular target for OA treatment.

## Materials and methods

### OA patient samples

Human cartilage tissues were obtained from patients with OA who underwent total knee arthroplasty. The Ethics Committee of Sir Run Run Shaw Hospital approved the experimental protocols. All patients provided written informed consent. After being stained with Safranin O/Fast Green, the human tissues were graded according to the Osteoarthritis Research Society International (OARSI) grading system^31^.

### Animal experiments

FTO global knockout (FTO^−/−^) and FTO conditionally knockout (FTO cKO) mice were purchased from Cyagen (Suzhou, China). FTO conditionally knockout (FTO cKO) mice were crossed with Col2-CreERT2 mice (Cyagen, China). A mouse direct PCR kit (Bimake, China) was used to confirm the genotypes of the mice. SLAC Laboratory Animal Company (Shanghai, China) supplied the WT C57BL/6 (B6) mice. The protocols followed during the study were approved by the Ethics Committee of Zhejiang University.

To create the DMM model, we transected the medial meniscotibial ligament in 12-week-old male C57BL/6 mice^32^. A sham operation was performed on control mice. The mice were euthanized 8 weeks after surgery. The Research Center of Regenerative Medicine, Guangxi Medical University, sacrificed spontaneous OA STR/Ort mice and control CBA/CaCrl mice at 6 months of age and donated them^33,34^. The OA model mice were evaluated on the basis of OARSI grade.

Animal praxeology assays, including the hot plate test and rotarod test, were performed on FTO cKO mice^35^. Before all of the behavioral experiments, the mice were acclimatized to the test room for 30 min.

The adeno-associated virus (AAV), purchased from HANBIO (Shanghai, China), was injected into the knee joint cavity of mice 1 week after DMM surgery at a dose of 5 × 10^9^ PFUs per 10 Μl using a 10 μL microsyringe with a 34 G needle (Hamilton Company, Reno, NV, USA).

### Immunohistochemistry

Tissues from mouse and human cartilage were fixed in 4% paraformaldehyde and decalcified in 0.5 M EDTA. They were decalcified before being embedded in paraffin and sectioned at 3 μm. The sections were then deparaffinized with xylene and hydrated with graded ethanol. Next, we treated some sections with 1% Fast/Green (Sigma‒Aldrich) for 3–5 min, 1% acetic acid for 10 s, and 1% Safranin O (Sigma‒Aldrich) for 3–5 min. For immunohistochemistry, after deparaffinization and hydration, the sections were treated with 3% hydrogen peroxide (H_2_O_2_) and 5% BSA and incubated overnight with the indicated antibodies for immunohistochemistry. The sections were then treated with horseradish peroxidase (HRP)-conjugated secondary antibodies. Finally, the sections were stained with DAB (Sigma Aldrich) and hematoxylin (Beyotime). A KFBIO scan and analysis system (KFBIO, Zhejiang, China) was used to scan the sections.

### Cell culture

We isolated mouse chondrocytes from 5-day-old WT, FTO^+/−^, FTO^fl/fl^, and the indicated control mice using 0.2% collagenase digestion. Dulbecco’s modified Eagle’s medium (DMEM; Gibco, Amarillo, TX, USA) supplemented with 10% fetal bovine serum (FBS; Gibco) and 1% penicillin/streptomycin was used as the culture medium. The first passage of chondrocytes was used for analysis. Human articular chondrocytes were isolated from the tibial plateaus and femoral condyles of human cartilage and cultured in the same manner as the mouse chondrocytes. Passage 0 chondrocytes were used for analysis. C28/I2 normal chondrocytes were supplied by Crisprbio (Beijing, China), and the American Type Culture Collection supplied HEK 293 T cells. Both strains were cultured in DMEM supplemented with 10% FBS (Gibco) and 1% penicillin G and streptomycin. The cells were cultured in a humid environment with 5% CO_2_ and 95% air.

HANBIO (Shanghai, China) and Tsingke (Beijing, China) supplied plasmids and lentiviruses. Chondrocytes were seeded in 6-well plates, and 1 mL of culture medium was added to each well. The chondrocytes were then transfected with FTO or SMAD2 lentivirus (1 × 10^7^ PFUs) and incubated for 12 h. Next, the wells were filled with 1 mL of culture medium and polybrene (Yeasen, Shanghai, China) was added at a final concentration of 10 μg/mL, and they were incubated for another 36 h. Finally, the original medium was replaced with fresh culture medium.

Primary adenovirus was used to transfect the chondrocytes of FTO^fl/fl^. Chondrocytes were seeded in 6-well plates, and 1 mL of culture medium was added. The Cre adenovirus (5 × 10^7^ PFUs) was then added to the medium at a density of approximately 50%, and the cells were incubated for 4 h. The wells were then filled with another 1 mL of culture medium and incubated for another 4–6 h. Finally, the original medium was replaced with fresh culture medium.

### Micro-CT analysis

The mouse knee joints were dissected and fixed in 4% paraformaldehyde. The CT scan was run using a high-resolution μCT (SHIMADZU, Kyoto, Japan) with a 50-kVp voltage, 200-μA current, and 5.7 μm resolution per pixel. Following the same protocol and conditions, we then used vgstudiomax 34 software to perform 3-D reconstructions of the scanning data, including sagittal, transverse, and coronal sections. All of the samples were blinded and evaluated.

### Micromass culture and chondrocyte 3D agarose culture

According to previous studies, micromass cultures can be used to assess ECM deposition^36,37^. Approximately 150,000 primary mouse chondrocytes were seeded at the center of 12-well plates. The chondrocytes were fixed with 4% paraformaldehyde after 7 days of culture. At a pH of 0.2, the chondrocytes were stained with Alcian blue 8 GS (Solarbio, China). We used ImageJ software (version 1.53 C; National Institute of Health, Bethesda, MD, USA) to quantify the ECM deposition.

Chondrocyte 3D agarose culture was also used to evaluate the ECM deposition as described in a previous study^38^. To obtain a concentration of 2% agarose, four volumes of 2.5% agarose were mixed with one volume of 5× culture medium and 1× culture medium containing 10% FBS, 5% HEPES, and 5% penicillin/streptomycin. The cell suspension was mixed with the medium until a final concentration of 2 × 10^6^ cells/mL was obtained. Next, approximately 700 μL of the mixture was added to a 24-well plate, and the mixture was left for approximately 15 min until gelling. The culture medium was then added to each well and replaced daily. The hydrogels were treated with 4% paraformaldehyde after 14 d of culture, embedded in paraffin, sectioned at 7 μm, and stained with Alcian blue. The hydrogel was then embedded in paraffin, sectioned at 7 μm, and stained with Alcian blue.

### Western blot analysis and antibodies

To lyse the cells and obtain protein, we used RIPA lysis buffer (Fudebio, Hangzhou, China) mixed with protease inhibitor cocktails (Fudebio). First, protein concentrations were determined via the Fudebio Bicinchoninic Acid protein assay kit. After 45 min of SDS‒PAGE, equivalent amounts of protein were transferred to 0.22-μm PVDF membranes (Merck KGaA) at 290 mA for 100 min. The membranes were then blocked for 1 h with 5% BSA. The membranes were then incubated with high-affinity antibodies. Next, the membranes were washed with TBST and then incubated with horseradish peroxidase-conjugated secondary antibodies (FDM007 and FDR007, Fudebio). Finally, an enhanced chemiluminescence kit (FD8030, Fudebio) was used to detect the antibody signals.

The following antibodies were used for Western blot analysis: anti-β-actin (3700, Cell Signaling Technology [CST], Danvers, MA, USA), anti-MMP13 (ab39032, Abcam), anti-COL2A1 (M2139, Santa Cruz Biotechnology, Dallas, TX, USA), anti-Aggrecan (C8035, Millipore, Burlington, MA, USA), anti-MMP3 (ab52915, Abcam), anti-SOX9 (ab185966, Abcam), anti-FTO (27226-1-AP, Proteintech, Rosemont, IL, USA), anti-SMAD2 (ab33875, Abcam), anti-RUNX1 (ab23980, Abcam), and anti-YTHDF2 (ab220163, Abcam) antibodies.

### RNA isolation and RT‒PCR

Total RNA was extracted from cells using the RNAEX reagent (Accurate Biotechnology, Hunan, China) and the SteadyPure Universal RNA Extraction Kit (Accurate Biotechnology) according to the manufacturer’s instructions. cDNA was generated through reverse transcription via the use of Evo M-MLV RT Premix for qPCR (Accurate Biotechnology). Then, using HieffR qPCR SYBR Green Master Mix (Yeasen, Shanghai, China), we performed RT‒PCR on a QuantStudio^TM^ 6 Flex Real-Time PCR System (Thermo Fisher Scientific, USA) according to the manufacturer’s instructions. The mRNA housekeeping gene β-actin was used as an internal control. Tsingke (Beijing, China) supplied the primers.

### RNA m6A dot blot assay

Poly(A)^+^ mRNAs extracted from human and mouse primary chondrocytes were spotted onto nylon membranes (GE Healthcare). After UV cross-linking (254 nm) and blocking, the membrane was incubated with an m6A-specific antibody (Cell Signaling Technology). The membrane was then exposed to a visualizer (GE Healthcare). Methylene blue was used to stain other poly(A)+ mRNAs spotted on the membrane.

### RNA m6A quantification

Total RNA was extracted from cells using the RNAEX reagent (Accurate Biotechnology, Hunan, China) and the SteadyPure Universal RNA Extraction Kit (Accurate Biotechnology) according to the manufacturers’ instructions. A NanoDrop3000 was used to assess RNA quality. An EpiQuik m6A RNA Methylation Quantification Kit (Colorimetric) (P-9005, Epigentek, USA) was used to measure the m6A content of total RNA. The capture and detection antibody solutions were added to each well after 200 ng of RNA was added per well according to the manufacturer’s instructions. The absorbance of each well at 450 nm was used to quantify the m6A levels in each well colorimetrically. The values were then calculated using a standard curve.

### mRNA stability

Actinomycin D (MCE, China, 2 μg/mL) inhibited further RNA synthesis in FTO^fl/fl^ primary chondrocytes cultured and transfected with Cre adenovirus or vector. Total RNA was extracted from chondrocytes every 2 h after treatment began. The remaining SMAD2 mRNA levels were calculated using quantitative RT‒PCR and normalized to those of the first group (0 h).

### Cell counting Kit-8 (CCK-8) assay

Normal primary chondrocytes and primary chondrocytes from FTO^fl/fl^ cells were cultured and transfected with Cre adenovirus or vector before being seeded into a 96-well plate. Normal primary chondrocytes were treated with FB23-2 at three different concentrations. The normal cell medium was replaced after 48 h with fresh medium containing 10% CCK-8 solution (Yeasen, Shanghai, China) and incubated at 37 °C for 1–4 h. The OD 450 nm of each well was measured using the Multiskan^TM^ FC System (Thermo Fisher Scientific).

### Flow cytometry assay

The intracellular ROS levels in primary chondrocytes were measured using a reactive oxygen species assay kit (Beyotime, Shanghai, China). We added DCFH-A to the culture medium and incubated it at 37 °C for 20 min, according to the manufacturer’s instructions. Following incubation, FITC-A was detected via a BD FACS flow cytometer (BD Biosciences).

### Luciferase reporter assays

The region upstream −2000 bp of the promoter of FTO was inserted into a pGL3-BASIC vector (HANBIO, Shanghai, China). Then, the HEK 293 T cells were placed into 24-well plates and transfected with a specific luciferase reporter plasmid using Lipofectamine 3000 transfection reagent (Thermo Fisher Scientific, USA) according to the manufacturer’s instructions. After 24 h of incubation, we used a dual-luciferase reporter assay system (Promega, Madison, WI, USA) to measure luciferase activity and calculated the relative luciferase activity using the value of hRLuc standardized to hFLuc.

### Chromatin immunoprecipitation assay (ChIP)

A Bersinbio ChIP kit (Bersinbio, China) was used to perform the ChIP assay according to the manufacturer’s instructions. First, 1% formaldehyde was used in the process of extracting DNA from primary mouse chondrocytes. The DNA was then sheared to the optimal fragment length via ultrasonication and confirmed using agarose gel electrophoresis. Next, the samples in the input and IP groups were collected with magnetic beads. After the IP mixtures were incubated overnight with anti-RUNX1 antibodies (ab23980; Abcam, Cambridge, UK), specific DNAs were screened using A/G magnetic beads. Finally, the immunoprecipitated DNA fragments were analyzed via quantitative RT‒PCR.

### m6A-RNA immunoprecipitation (MeRIP) assay

We used the RNAEX reagent (Accurate Biotechnology, Hunan, China) to extract total RNA from primary chondrocytes of FTO^fl/fl^ cells cultured and transfected with the Cre adenovirus or vector. Following the instructions for inactivation of the methylated RNA immunoprecipitation (MeRIP) m6A kit (Bersinbio, China), the RNAs were incubated with an anti-m6A antibody (ab284130, Abcam, Cambridge, UK) for immunoprecipitation. qRT‒PCR was used to analyze m6A mRNA enrichment.

### RNA binding protein immunoprecipitation (RIP) assay

We used an RNA-binding protein immunoprecipitation (RIP) assay kit (Bersinbio, China) to perform RIP analysis according to the manufacturer’s instructions. Primary chondrocytes from FTOfl/fl cells that had been cultured and transfected with Cre adenovirus or vector were treated with protease and RNase inhibitors before being incubated overnight with an anti-YTHDF2 antibody (ab220163, Abcam, Cambridge, UK). qRT‒PCR was used to examine the mRNAs screened by the antibody and magnetic beads.

### RNA sequencing

We used the RNAEX reagent (Accurate Biotechnology, Hunan, China) to extract total RNA from primary chondrocytes of FTO^fl/fl^ cells cultured and transfected with the Cre adenovirus or vector. LC-Bio Technology (Hangzhou, China) was used for reverse transcription into cDNA and PCR amplification. An Illumina NovaSeq 6000 (LC-Bio Technology, Hangzhou, China) was used to perform 2 × 150-bp paired-end sequencing (PE150). The significantly differentially expressed mRNAs were selected if the fold change range was >1.5 or < −1.5 and the *P*-value was < 0.01.

### RNA m6A sequence

RNAEx reagent (Accurate Biotechnology, Hunan, China) was used to extract total RNA from primary chondrocytes of FTO^fl/fl^ cells cultured and transfected with Cre adenovirus or vector. LC-Bio Technology (Hangzhou, China) was used to enrich the mRNA fragments by RIP and detect different m6A peaks. An Illumina NovaSeq 6000 (LC-Bio Technology, Hangzhou, China) was used to perform 2 × 150 bp paired-end sequencing (PE150). The significant differential m6A-modified peaks were those with a fold change > 0.5 and a *P*-value < 0.01.

### Statistics

The data in this study were analyzed via GraphPad Prism (version 8; San Diego, CA, USA) and are presented as the means ± SDs. Student’s t-test was used to compare significant differences between two groups, and one-way ANOVA was used to calculate differences between multiple groups. Statistical significance was set at *p < 0.05, **p < 0.01, ***p < 0.001, and ****p < 0.0001.

## Results

### FTO expression decreased in OA with increasing m6A levels

**Fig. 1:**
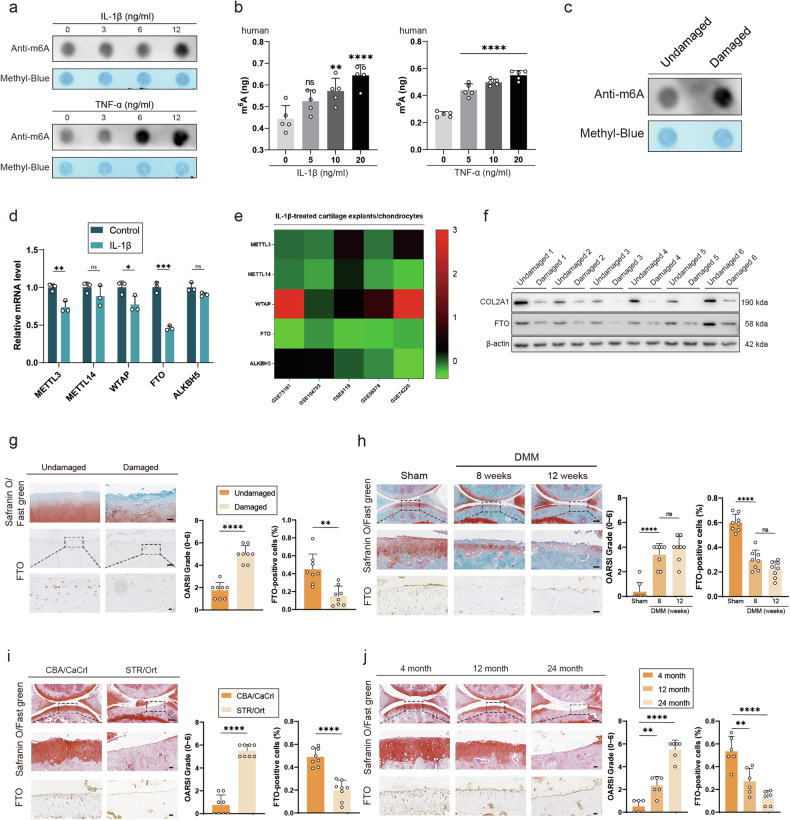
FTO expression decreases in OA with increasing m6A levels. **a** Dot blot of human primary chondrocytes treated with IL-1β or TNF-α. **b** Results from the m6A quantitative kit for human primary chondrocytes treated with IL-1β or TNF-α. **c** Dot blot of human primary chondrocytes from the damaged cartilage group or the undamaged cartilage group. **d** mRNA expression levels of METTL3, METTL14, WTAP, FTO and ALKBH5 in primary mouse chondrocytes treated with IL-1β. **e** Five databases related to IL-1β treatment. **f** Western blot detection of FTO expression in human primary chondrocytes from the damaged cartilage group or the undamaged cartilage group. **g** Safranin O/Fast Green staining and immunohistochemistry (FTO) of undamaged and damaged regions from the lateral and medial tibial plateaus of OA patients. *n* = 8. Scale bar, 100 μm and 20 μm. **h** Safranin O/Fast Green staining and immunohistochemistry (FTO) of the knee joints of mice 8 or 12 weeks after DMM surgery or in the sham group. *n* = 8 per group. Scale bars, 100 μm and 20 μm. **i** Safranin O/Fast Green staining and immunohistochemistry (FTO) of the knee joints of STR/Ort mice and CBA/CaCrt mice. *n* = 8 per group. Scale bars, 100 μm and 20 μm. **j** Safranin O/Fast Green staining and immunohistochemistry (FTO) of the knee joints of aged mice. *n* = 8 per group. Scale bars, 100 μm and 20 μm; the data are representative of three independent experiments (**f**). *p < 0.05, **p < 0.01, ***p < 0.001, ****p < 0.0001, (**d**, **g**, **h**, **i**) mean ± SD, two-tailed t test; (**b**, **j**) mean ± SD, one-way ANOVA.

To investigate the role of m6A in OA development, human or mouse chondrocytes were treated with interleukin-1β (IL-1β) or tumor necrosis factor α (TNF-α) to mimic the inflammatory environment during OA progression^5^. According to the results of the dot blot analysis, the m6A levels were greater in the longer treatment groups (Fig. 1a). Moreover, the m6A quantitative kit revealed that human or mouse chondrocytes in the group exposed to relatively high concentrations of IL-1β or TNF-α presented relatively higher levels of m6A (Fig. 1b and Supplementary Fig. 1a). In addition, m6A levels were greater in the damaged cartilage group than in the undamaged cartilage group (Fig. 1c). These findings revealed a positive correlation between OA and m6A levels.

Among the five most common m6A-related genes in primary mouse chondrocytes treated with IL-1β, the expression of two m6A “writers,” METTL3 and WTAP, and the expression of one m6A “eraser,” FTO, decreased significantly (Fig. 1d)^39^. In addition, five databases related to IL-1β treatment were analyzed for comparison with the RT‒PCR results. FTO expression was downregulated in response to IL-1β treatment (Fig. 1e).

In addition to investigating inflammation, we also investigated FTO expression in OA via oxidative stress. Compared with the undamaged cartilage group, the damaged cartilage group presented lower FTO expression (Fig. 1f). Moreover, FTO transcript and protein expression in mouse chondrocytes was lower in groups treated with higher concentrations of IL-1β, TNF-α, or hydrogen peroxide (Supplementary Fig. 1b–k). Changes in reactive oxygen species (ROS) levels were used to assess oxidative stress (Supplementary Fig. 1l). These findings suggest that FTO expression is reduced in response to inflammatory stimulation and oxidative stress. FTO expression was measured in human OA articular cartilage sections. The damaged group had higher OARSI scores and lower FTO expression levels (Fig. 1g). FTO expression was also measured in DMM-induced OA model mice. The DMM group had higher OARSI scores, whereas FTO expression was low (Fig. 1h). FTO expression was measured in STR/Ort mice, which spontaneously develop OA. The OARSI scores of the STR/Ort group were greater, whereas FTO expression was lower (Fig. 1i). The age-induced OA mouse model also had higher OARSI scores and lower FTO expression (Fig. 1j). These findings revealed that FTO expression was lower in human OA cartilage sections and mouse OA models.

### Reduced FTO contributed to the catabolic effects of chondrocytes in vivo and in vitro

**Fig. 2:**
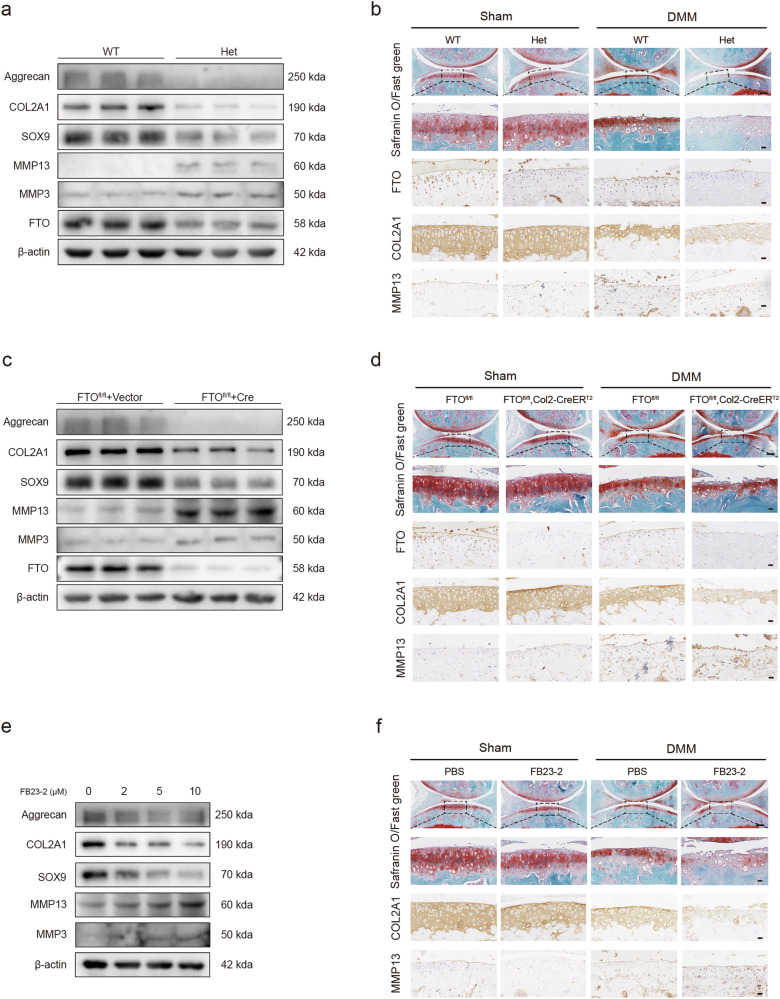
Reduced FTO contributed to the catabolic effects of chondrocytes in vivo and in vitro. **a** Western blot detection of FTO, COL2A1, Aggrecan, SOX9, MMP3 and MMP13 in primary chondrocytes isolated from FTO^+/+^ or FTO^+/−^ mice. **b** Safranin O/Fast Green staining and immunohistochemistry (FTO, COL2A1 and MMP13) of the knee joints of FTO^+/+^ or FTO^+/−^ mice that underwent DMM surgery or sham surgery. *n* = 8 per group. Scale bars, 100 μm and 20 μm. **c** Western blot detection of FTO, COL2A1, Aggrecan, SOX9, MMP3 and MMP13 in primary chondrocytes isolated from FTO-cKO mice cultured and transfected with Cre adenovirus or vector. **d** Safranin O/Fast Green staining and immunohistochemistry (FTO, COL2A1 and MMP13) of knee joints from FTO^fl/fl^ and FTO^fl/fl^, Col2a1-CreER^T2^ mice that underwent DMM surgery or sham surgery. *n* = 8 per group. Scale bars, 100 μm and 20 μm. **e** Western blot detection of COL2A1, Aggrecan, SOX9, MMP3 and MMP13 in a concentration-dependent manner in mouse chondrocytes treated with FB23-2. **f** Safranin O/Fast Green staining and immunohistochemistry (COL2A1 and MMP13) of the knee joints of the mice that underwent DMM surgery or the sham group. Articular injection of FB23-2 or PBS was performed weekly. *n* = 8 per group. Scale bars, 100 μm and 20 μm; the data are representative of three independent experiments (**a**, **c**, **e**).

We generated mice with a homozygous deletion of FTO to determine whether downregulated FTO affects the pathological progression of OA. However, homozygous deletion of FTO in mice (FTO^−/−^) is embryonically lethal^40^. Therefore, we evaluated the effects of FTO on heterozygous chondrocytes. We also created cartilage-specific conditional knockout (cKO) mice (FTO^fl/fl^, Col2a1-CreER^T2^). FTO expression decreased significantly in primary cultured chondrocytes isolated from FTO-cKO and FTO^+/−^ mice (Fig. 2a, c).

When FTO was knocked out in primary cultured chondrocytes isolated from FTO-cKO mice, the transcript and protein expression of catabolic enzymes, including MMP3 and MMP13, increased, whereas the expression of anabolic enzymes, including Aggrecan, COL2A1, and SOX9, decreased significantly (Fig. 2c and Supplementary Fig. 2c). These findings suggest that in the absence of FTO, catabolic enzyme expression increases, whereas anabolic enzyme expression decreases. The same pattern was observed in FTO^+/−^ mice (Fig. 2a and Supplementary Fig. 2a).

The mice were subjected to a DMM-induced osteoarthritis model. In the sham group, no significant difference in OARSI scores between the FTO^fl/fl^, Col2a1-CreER^T2^, and FTO^fl/fl^ groups was observed, whereas the FTO^fl/fl^, Col2a1-CreER^T2^ mice had higher OARSI scores than the FTO^fl/fl^ mice in the DMM group (Fig. 2d and Supplementary Fig. 2d). Immunohistochemistry (IHC) revealed that FTO^fl/fl^, Col2a1-CreER^T2^ mice had lower COL2A1 expression and higher MMP13 expression than FTO^fl/fl^ control mice did in the DMM group (Fig. 2d and Supplementary Fig. 2d). Moreover, the FTO^fl/fl^, Col2a1-CreER^T2^ mice presented more osteophytes (Supplementary Fig. 2e). In addition, FTO^fl/fl^, Col2a1-CreER^T2^ mice were more sensitive to pain and had some degree of mobility impairment in the hot plate test and rotarod test (Supplementary Fig. 2f). The Safranin O staining and IHC results of the heterozygous and wild-type mice were similar to those of the FTO-cKO mice, indicating that FTO knockdown resulted in decreased COL2A1 expression and increased MMP13 expression and OARSI scores (Fig. 2b and Supplementary Fig. 2b).

The FTO inhibitor FB23-2 was used to validate the role of FTO in OA. FTO proteins were rendered inactive in FB23-2-treated chondrocytes^41^. The Western blot and RT‒PCR results revealed the same trends as those in the heterozygous and cKO mice (Fig. 2e and Supplementary Fig. 3a). Micromass culture and 3D agarose culture of chondrocytes revealed that ECM deposition decreased in the groups treated with high concentrations of FB23-2 (Supplementary Fig. 3b, c). Safranin O staining and IHC further supported the findings from either FTO-cKO mice or heterozygotes (Fig. 2f and Supplementary Fig. 3d). Moreover, Western blotting and RT‒PCR revealed that FB23-2 did not affect the expression of FTO but did affect its activity (Supplementary Fig. 3e, f). In addition, the CCK8 assay results revealed that FB23-2 did not affect cell viability (Supplementary Fig. 3g). The viability of primary cultured chondrocytes isolated from FTO^fl/fl^ mice treated with Cre adenovirus or the vector did not differ statistically (Supplementary Fig. 3h). These findings indicated that changes in the expression of catabolic and anabolic enzymes in primary cultured chondrocytes isolated from FTO-cKO mice were due to differentiation dysfunction rather than proliferation.

### Runx1 regulated FTO gene transcriptional activity during OA progression

**Fig. 3:**
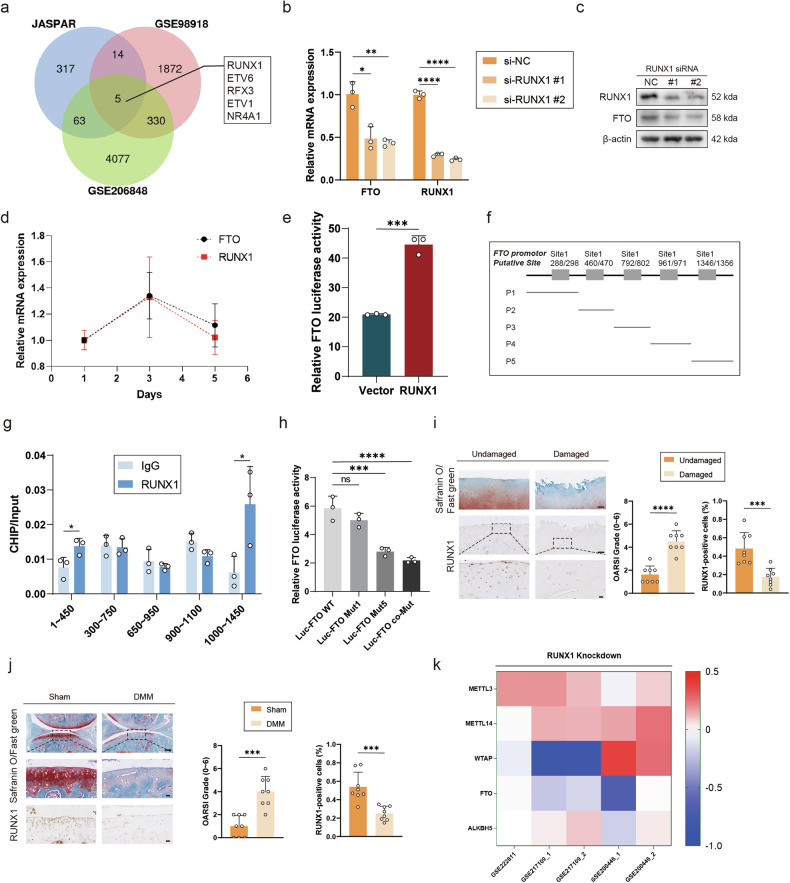
Runx1 regulated FTO gene transcriptional activity during OA progression. **a** Venn diagram showing the overlaps between transcription factors predicted by JASPAR and two OA-related databases, GSE98918 and GSE206848. **b** mRNA expression levels of FTO and RUNX1 in mouse chondrocytes treated with siRNAs targeting RUNX1. **c** Western blot detection of FTO and RUNX1 expression in mouse chondrocytes treated with siRNAs targeting RUNX1. **d** mRNA expression levels of FTO and RUNX1 on days 1, 3, and 5 of cell culture in mouse chondrocytes. **e** Luciferase activity detection of HEK293T cells transfected with RUNX1 (or vector) and the FTO reporter. **f** Five primer pairs designed on the basis of the prediction of JASPAR. **g** ChIP analysis of five sites on the FTO promoter in mouse chondrocytes transfected with RUNX1 or IgG. **h** Luciferase activity detection of HEK293T cells transfected with the FTO reporter or the FTO mut reporter. **i** Safranin O/Fast Green staining and immunohistochemistry (RUNX1) of undamaged and damaged regions from the lateral and medial tibial plateaus of OA patients. *n* = 8. Scale bar, 100 μm and 20 μm. **j** Safranin O/Fast Green staining and immunohistochemistry (RUNX1) of the knee joints of the mice that underwent DMM surgery or the sham group. *n* = 8 per group. Scale bars, 100 μm and 20 μm. **k** Five databases for RUNX1 knockdown; the data are representative of three independent experiments (**c**). *p < 0.05, **p < 0.01, ***p < 0.001, ****p < 0.0001, mean ± SD, two-tailed t test.

We used the online software JASPAR and two OA-related databases, GSE98918 and GSE206848, and screened 5 potential transcription factors that may regulate FTO transcription (Fig. 3a). Among them, the variation trend of NR4A1 expression was the opposite in the two databases, so it was excluded. Small interfering RNAs (siRNAs) targeting 4 transcription factors were used to treat primary mouse chondrocytes. FTO expression decreased by more than 50% after RUNX1 knockdown but by no more than 50% after other transcription factors were knocked down (Supplementary Fig. 4h). These findings suggest that RUNX1 is the transcription factor that most likely regulates FTO expression. In addition, two siRNAs targeting RUNX1 were administered to primary mouse chondrocytes. FTO expression was significantly decreased following treatment with the two small interfering RNAs, indicating that RUNX1 could regulate FTO expression (Fig. 3b, c). In addition, RT‒PCR revealed similar changes in FTO and RUNX1 expression on days 1, 3, and 5 of cell culture (Fig. 3d).

A luciferase assay was used to investigate the transcriptional regulation of FTO by RUNX1. The relative luciferase activity of FTO increased significantly after RUNX1 overexpression, indicating that RUNX1 overexpression enhanced FTO transcription (Fig. 3e).

JASPAR was used to predict the binding sites where RUNX1 binds to the FTO promoter. Five primer pairs were designed on the basis of this prediction (Fig. 3f). RUNX1 was significantly enriched at sites 1 and 5 in ChIP analysis (Fig. 3g). The mutant and co-mutant plasmids at sites 1 and 5 were designed and used in conjunction with luciferase (Supplementary Fig. 4i). FTO expression decreased significantly at site 5 compared with that in the wild-type group, suggesting that RUNX1 regulated FTO expression by binding to site 5 on the FTO promoter (Fig. 3h). Furthermore, to explore changes in RUNX1 expression in OA, primary mouse chondrocytes were treated with IL-1β, TNF-α or oxidative stress stimulation to mimic the environment of OA. RUNX1 expression was significantly decreased after treatment with the three different stimuli, as determined by Western blotting and RT‒PCR (Supplementary Fig. 4a‒g). In addition, IHC revealed that the damaged group had higher OARSI scores and lower RUNX1 expression, similar to the results from the DMM group (Fig. 3i, j).

To identify the general transcriptional regulators of FTO in different tissues, we used bioinformatics analysis of three other leukemia-related databases, GSE222811, GSE217109 and GSSE200446, to observe the expression of five enzymes associated with m6A. FTO expression was downregulated in response to RUNX1 knockdown (Fig. 3k), which was consistent with the results of OA.

### FTO-mediated SMAD2 m6A modification in a YTHDF2-dependent manner

**Fig. 4:**
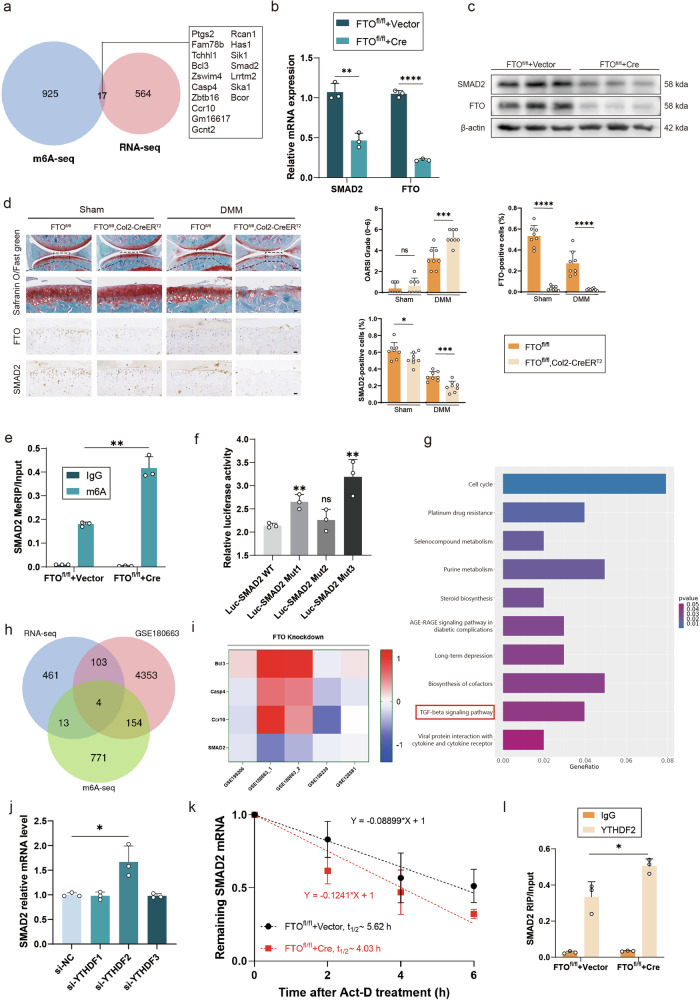
FTO mediated SMAD2 m6A modification in a YTHDF2-dependent manner. **a** Venn diagram showing the overlap between m6A-seq and RNA-seq data from primary chondrocytes isolated from FTO-cKO mice cultured and transfected with Cre adenovirus or vector. **b** mRNA expression levels of FTO and SMAD2 in primary chondrocytes isolated from FTO-cKO mice cultured and transfected with Cre adenovirus or vector. **c** Western blot detection of FTO and SMAD2 in primary chondrocytes isolated from FTO-cKO mice cultured and transfected with Cre adenovirus or vector. **d** Safranin O/Fast Green staining and immunohistochemistry (FTO and SMAD2) of the knee joints of FTO^fl/fl^ and FTO^fl/fl^, Col2a1-CreER^T2^ mice that underwent DMM surgery or sham surgery. *n* = 8 per group. Scale bars, 100 μm and 20 μm. **e** MeRIP analysis of SMAD2 in primary chondrocytes isolated from FTO-cKO mice cultured and transfected with Cre adenovirus or vector. **f** Luciferase activity detection of HEK293T cells transfected with the SMAD2 reporter or the SMAD2 mut reporter. **g** KEGG analysis of the GSE180663 dataset and transcriptome sequencing data. **h** Venn diagram showing the overlaps between transcription factors predicted by RNA-seq m6A-seq and GSE180663. **i** Results from five databases on FTO knockdown. **j** mRNA expression levels of SMAD2 in mouse chondrocytes treated with siRNAs targeting YTHDF1, YTHDF2 or YTHDF3. **k** The mRNA expression level of SMAD2 after treatment with actinomycin D at the indicated time points. **l** RIP analysis of the interaction of YTHDF2 with SMAD2 mRNA in primary chondrocytes isolated from FTO-cKO mice cultured and transfected with Cre adenovirus or vector. The data are representative of three independent experiments (**c**). *p < 0.05, **p < 0.01, ***p < 0.001, ****p < 0.0001, mean ± SD, two-tailed t test.

m6A sequencing and transcriptome sequencing were used to examine m6A modification changes and transcript expression changes in the primary chondrocytes of FTO^fl/fl^ cells cultured and transfected with the Cre adenovirus or vector to investigate the downstream molecular mechanism of FTO in OA. Significant changes in m6A modifications and transcript expression were detected in 17 genes (Fig. 4a and Supplementary Fig. 5a). In addition, RT‒PCR revealed that the expression of 11 genes changed significantly after treatment with IL-1β (Supplementary Fig. 5b). Only 3 genes showed statistically significant differences in expression between primary chondrocytes cultured from FTO^fl/fl^ transfected with Cre adenovirus or vector (Supplementary Fig. 5c). Among those genes, SMAD2 expression changed significantly and had the largest range of changes. Several previous studies have shown that SMAD2 is essential for OA protection^11,30,42^. Therefore, SMAD2 may be a downstream target of FTO in OA.

Western blotting and RT‒PCR were used to explore the effects of FTO on SMAD2 expression. SMAD2 expression decreased when FTO was knocked out (Fig. 4b, c). IHC also revealed that SMAD2 expression was lower in FTO-cKO mice than in control mice (Fig. 4d). MeRIP revealed that m6A levels on SMAD2 were greater in FTO^fl/fl^ mouse primary chondrocytes treated with Cre adenovirus than in those treated with the vector (Fig. 4e). These findings suggest that alterations in FTO expression may affect SMAD2 expression. Moreover, to explore the relationship between SMAD2 and OA, Western blotting was used, and the results revealed that SMAD2-knockdown chondrocytes presented decreased protein expression of SOX9 and ECM components and increased protein expression of catabolic enzymes, including MMP3 and MMP13 (Supplementary Fig. 6).

m6A sequencing was used to predict the three most likely m6A modification sites in SMAD2 mRNA to investigate the specific sites of m6A modification further. Three mutant plasmids were designed (Supplementary Fig. 5d). The plasmids were then transfected into primary chondrocytes, and the luciferase activity of SMAD2 was measured using a luciferase assay. These findings revealed that the luciferase activity of SMAD2 increased significantly in chondrocytes transfected with the mutant plasmid with site 1 or site 3 mutations compared with that in chondrocytes transfected with the wild-type plasmid, suggesting that sites 1 and 3 are m6A modification sites on SMAD2 mRNA (Fig. 4f).

The general molecular function of FTO in different tissues was also investigated. Bioinformatics analysis was performed on the thyroid cell-related database GSE199206, the liver cancer-related database GSE180663, the bladder cancer cell-related database GSE150239 and the breast cancer cell-related database GSE128581, and the expression of 17 genes previously obtained by m6A sequencing and transcriptome sequencing was observed (Supplementary Fig. 5e). Among the 17 genes, 12 could be analyzed. SMAD2 expression was downregulated in response to low FTO expression. KEGG analysis of GSE180663 revealed that genes related to the TGF-beta signaling pathway presented the most significant decline (Supplementary Fig. 5f). After the intersection of the GSE180663 dataset and the transcriptome sequencing data, the results of the KEGG analysis revealed that genes associated with the TGF-beta signaling pathway were still among the top ten pathways with the most significant decline in expression (Fig. 4g). Previous studies have shown that SMAD2 is an important downstream molecule in the TGF-beta signaling pathway^43^. At the intersection of m6A sequencing, transcriptome sequencing and GSE180663, four differentially expressed genes were obtained, including Bcl3, Casp4, Ccr10 and SMAD2, among which only the expression of SMAD2 was reduced after the decrease in FTO expression (Fig. 4h, i). These results indicate that low expression of FTO in different tissues can lead to a decrease in SMAD2 expression.

While FTO serves as an eraser for m6A on SMAD2, m6A-selective binding proteins are required to recognize m6A-modified mRNAs and perform regulatory functions. Primary chondrocytes were treated with three different types of small interfering RNAs corresponding to three common “readers,” including YTHDF1/2/3, to knock them down. Among them, SMAD2 mRNA expression increased significantly only after YTHDF2 knockdown (Fig. 4j). YTHDF2 recognizes mRNAs that have been modified by m6A, which reduces its stability^39^. Actinomycin D was also used to treat FTO^fl/fl^ primary chondrocytes (transfected with Cre adenovirus or vector), as well as primary mouse chondrocytes (transfected with si-YTHDF2 or si-NC). RT‒PCR revealed that SMAD2 mRNA degradation was faster in FTO^fl/fl^ chondrocytes transfected with Cre adenovirus than in those transfected with the vector; it was slower in primary mouse chondrocytes transfected with si-YTHDF2 than in those transfected with si-NC, indicating that SMAD2 mRNA degraded faster in chondrocytes lacking FTO and more slowly in chondrocytes lacking YTHDF2 (Fig. 4k and Supplementary Fig. 5i). RIP analysis revealed that YTHDF2 was more enriched in SMAD2 mRNA in FTO^fl/fl^ primary chondrocytes cultured and transfected with the Cre adenovirus than in chondrocytes treated with the vector (Fig. 4l). These findings revealed that YTHDF2 plays an important role in the effect of FTO on SMAD2 mRNA expression.

Another two YTHDF2 small interfering RNAs were designed and used to treat FTO^fl/fl^ primary chondrocytes cultured and transfected with Cre adenovirus. After treatment with small interfering RNAs targeting YTHDF2, the transcript and protein expression of anabolic enzymes, including Aggrecan, COL2A1, and SOX9, increased significantly, whereas the expression of catabolic enzymes, including MMP3 and MMP13, did not significantly change, as revealed via Western blotting and RT‒PCR, indicating that the decrease in YTHDF2 expression influenced the expression of anabolic enzymes (Supplementary Fig. 5g, h).

### Overexpressing FTO or SMAD2 partially alleviated OA progression in the DMM mouse model

**Fig. 5:**
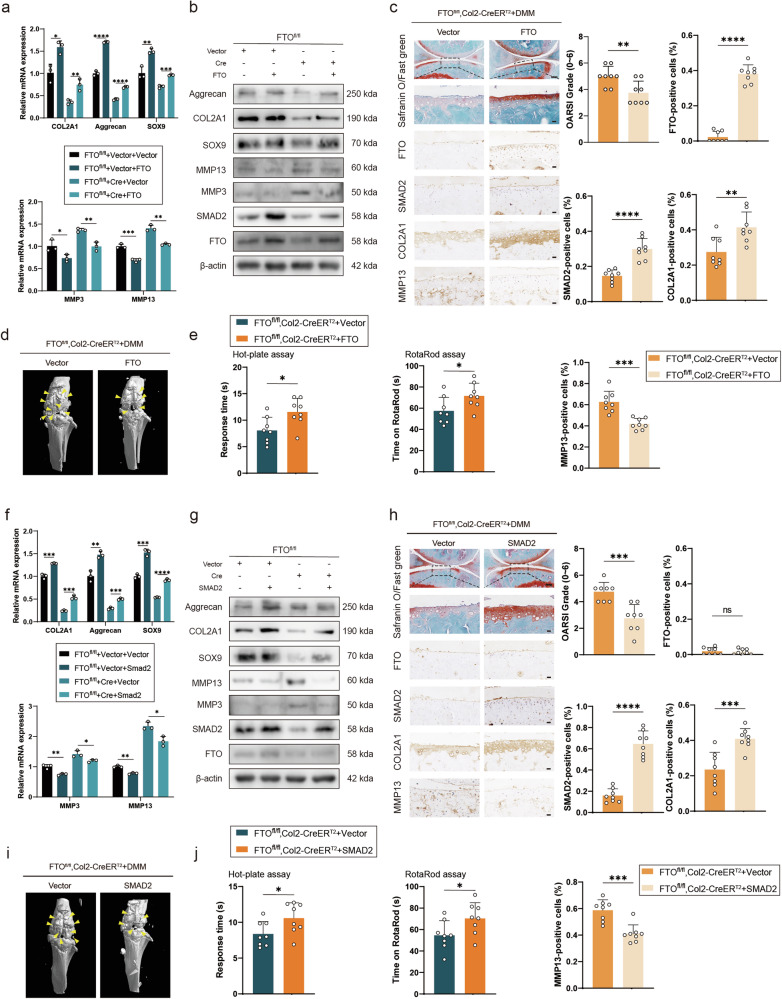
Overexpressing FTO or SMAD2 partially alleviated OA progression in the DMM mouse model. **a** mRNA expression levels of COL2A1, Aggrecan, SOX9, MMP3 and MMP13 in primary chondrocytes isolated from FTO-cKO mice cultured and transfected with Cre adenovirus (or vector) and FTO (or vector). **b** Western blot detection of FTO, SMAD2, COL2A1, Aggrecan, SOX9, MMP3 and MMP13 in primary chondrocytes isolated from FTO-cKO mice cultured and transfected with Cre adenovirus (or vector) and FTO (or vector). **c** Safranin O/Fast Green staining and immunohistochemistry (FTO, SMAD2, COL2A1 and MMP13) of the knee joints of FTO^fl/fl^, Col2a1-CreER^T2^ mice that underwent DMM. Articular injection of FTO adeno-associated virus (AAV) or vector. *n* = 8 per group. Scale bars, 100 μm and 20 μm. **d** Micro-CT images of the knee joints of FTO-cKO mice that underwent DMM surgery. Articular injection of FTO adeno-associated virus (AAV) or vector. Yellow arrows indicate the osteophytes. **e** The hot plate and rotarod test results of FTO-cKO mice that underwent DMM surgery. Articular injection of FTO adeno-associated virus (AAV) or vector. **f** mRNA expression levels of COL2A1, Aggrecan, SOX9, MMP3 and MMP13 in primary chondrocytes isolated from FTO-cKO mice cultured and transfected with Cre adenovirus (or vector) and SMAD2 (or vector). **g** Western blot detection of FTO, SMAD2, COL2A1, Aggrecan, SOX9, MMP3 and MMP13 in primary chondrocytes isolated from FTO-cKO mice cultured and transfected with Cre adenovirus (or vector) and SMAD2 (or vector). **h** Safranin O/Fast Green staining and immunohistochemistry (FTO, SMAD2, COL2A1 and MMP13) of the knee joints of FTO^fl/fl^, Col2a1-CreER^T2^ mice that underwent DMM. Articular injection of SMAD2 adeno-associated virus (AAV) or vector. *n* = 8 per group. Scale bars, 100 μm and 20 μm. **i** Micro-CT images of the knee joints of FTO-cKO mice that underwent DMM surgery. Articular injection of SMAD2 adeno-associated virus (AAV) or vector. Yellow arrows indicate the osteophytes. **j** The hot plate and rotarod test results of FTO-cKO mice that underwent DMM surgery. Articular injection of SMAD2 adeno-associated virus (AAV) or vector. The data are representative of three independent experiments (**b**, **g**). *p < 0.05, **p < 0.01, ***p < 0.001, ****p < 0.0001, mean ± SD, two-tailed t test.

We designed an FTO-overexpressing lentiviral plasmid and transfected it into the primary chondrocytes of FTO^fl/fl^ cells, which were subsequently cultured and transfected with Cre adenovirus or vector to confirm whether it could suppress OA progression. FTO expression increased after treatment with the FTO overexpression lentiviral plasmid, as shown by Western blotting (Fig. 5b). Western blotting and RT‒PCR analysis of FTO^fl/fl^ primary chondrocytes (transfected with Cre adenovirus) transfected with the FTO lentivirus or vector revealed that the transcript and protein expression of catabolic enzymes, including MMP3 and MMP13, decreased in response to the overexpression of FTO. In contrast, the levels of anabolic enzymes, including Aggrecan, COL2A1, and SOX9, increased significantly after treatment with the FTO lentivirus. SMAD2 expression also increased after treatment with the FTO lentivirus (Fig. 5a, b). In addition, after DMM surgery, FTO adeno-associated virus (AAV) was injected into the articular cavity of FTO^fl/fl^, Col2-CreER^T2^ mice. OARSI scores were lower in the group treated with FTO AAV than in the group treated with vector, as determined by Safranin O staining (Fig. 5c). In addition, IHC revealed that the expression of Aggrecan, COL2A1, SOX9 and SMAD2 was greater in the group treated with FTO AAV, whereas the expression of MMP13 and MMP3 was lower, indicating that the decrease in the expression of catabolic enzymes, including MMP13 and MMP3, and the increase in the expression of Aggrecan, COL2A1, SOX9 and SMAD2 were related to the increase in FTO in DMM-induced OA model mice in the FTO^fl/fl^, Col2-CreER^T2^ mice (Fig. 5c and Supplementary Fig. 7). In addition, the FTO^fl/fl^, Col2a1-CreER^T2^ mice treated with FTO AAV had fewer osteophytes (Fig. 5d). FTO^fl/fl^, Col2a1-CreER^T2^ mice treated with FTO AAV were less sensitive to pain and had a lower degree of mobility impairment in the hot plate test and rotarod test (Fig. 5e).

A lentiviral plasmid overexpressing SMAD2 was then designed and transfected into primary chondrocytes of FTO^fl/fl^ cells cultured and transfected with Cre adenovirus or vector to investigate whether it influences OA progression. The Western blotting and RT‒PCR results were comparable to those of primary chondrocytes treated with FTO lentivirus. However, FTO expression did not significantly differ after treatment (Fig. 5f, g). Except for a significant difference in FTO expression, the results of Safranin O staining and IHC were similar to those after FTO AAV treatment (Fig. 5h). The results of the micro-CT and animal praxeology assays were also similar to those after FTO AAV treatment (Fig. 5i, j). These findings indicated that the decrease in catabolic enzymes and the increase in anabolic enzymes were related to the increase in SMAD2 and that SMAD2 expression did not affect FTO expression changes in DMM-induced OA mouse models of FTO^fl/fl^, Col2-CreER^T2^ mice. These findings suggest that FTO and SMAD2 are potential therapeutic targets for OA.

## Discussion

Osteoarthritis (OA) is a common degenerative disease with a significant social and economic burden^44^. N6-methyladenosine (m6A) is a common RNA posttranscriptional modification that affects mRNA stability, splicing, transport, localization, and translation efficiency^45,46^. Several studies have shown that m6A plays an important regulatory role in neurons and tumors. METTL3 can influence liver development^47^ and HCC progression^48^ and regulate CRC aggressiveness and metastasis^49^. FTO regulates BNIP3 to influence tumor growth and metastasis in breast cancer^50^. FTO regulates Ehmt2 and G9a in the dorsal root ganglion, which affects neuropathic pain (DRG)^51^. However, the role of m6A in OA remains unclear. Our study revealed that m6A is associated with OA progression. We found that the m6A modification level was greater in the joint tissue of patients with OA than in normal tissue and was greater in the joint tissue of mice treated with DMM than in that of the sham group. Our findings revealed that m6A modification levels are positively correlated with OA severity.

Primary studies have focused on the relationship between FTO and OA. FTO overexpression regulates miR-515-5p expression to alleviate OA^27^. In addition, AC008440.5 may be regulated by FTO during OA progression^28^. These studies confirmed that FTO plays an important role in alleviating OA through in vitro data. In our study, we found that the demethylation enzyme FTO is downregulated during OA progression and plays an important role in the relationship between m6A and OA in vivo. In this study, we constructed heterozygous FTO-cKO mice. COL2A1, Aggrecan, and SOX9 protein expression was significantly decreased in primary chondrocytes with low FTO expression or without FTO, whereas MMP3 and MMP13 expression was increased. IHC analysis of mouse joint tissues from the DMM or sham groups revealed that COL2A1 expression decreased while MMP13 expression increased after DMM treatment. We also found that when chondrocytes were treated with FB23-2, an FTO inhibitor, anabolism was weakened, while catabolism was increased. Articular injection of FB23-2 into the joint cavity of wild-type mice yielded comparable results to those of FTO^+/−^ and FTO-cKO mice. Moreover, the regulatory role of FTO in OA is not sufficiently clear.

RUNX1 is a transcription factor that plays an important role in OA^29^. Previous studies have revealed that the overexpression of RUNX1 can help alleviate OA^7^. These studies are consistent with our findings. Our findings revealed that RUNX1 affects FTO expression in OA via luciferase and ChIP assays. In addition, the protein expression levels of RUNX1 and FTO follow similar patterns during OA progression. These findings suggest that, as a transcription factor in OA, RUNX1 regulates FTO expression by binding to specific sites on the FTO promoter. RUNX1 was previously shown to regulate FTO expression in the DRG to induce neuropathic pain, suggesting that RUNX1 could regulate FTO expression in multiple settings^51^. However, there may be other ways to regulate FTO in addition to transcriptional regulation, which requires further investigation.

SMAD2 is a key protein in the regulation of OA and plays a critical role in regulating anabolism and catabolism^42,52^. It is involved in the process of TGF-β regulation in OA. Previous studies have confirmed that TGF-β and SMAD2 can alleviate OA, suggesting that SMAD2 is a potential therapeutic target for OA^9^. However, the role of FTO in regulating SMAD2 expression in OA has not been thoroughly investigated. In our study, we used m6A sequencing and transcriptome sequencing to determine whether FTO alters the m6A levels of SMAD2 mRNA. MeRIP and luciferase assays revealed that FTO could change m6A modifications at specific sites on SMAD2 mRNA. YTHDF2 plays an important role in m6A, as it recognizes m6A modifications and subsequently reduces mRNA stability^22^. RIP analysis revealed that FTO regulates SMAD2 expression through YTHDF2. However, we cannot rule out the possibility that FTO may also play regulatory roles in OA via other mechanisms, possibly through different “m6A readers,” necessitating further studies to investigate this possibility.

**Fig. 6:**
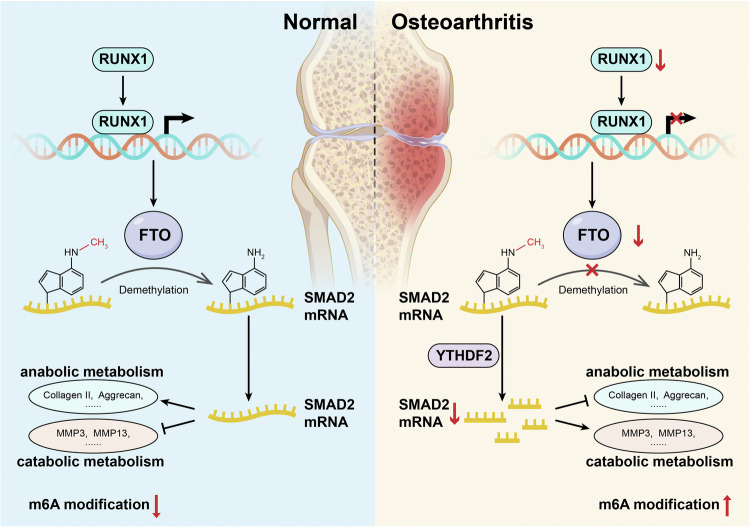
Schematic diagram of how FTO-mediated SMAD2 m6A modification protects cartilage against osteoarthritis. In human OA samples, m6A modification increased, whereas the expression of the demethylase FTO decreased. The dysfunction of RUNX1-mediated FTO transcription was attributed to decreased FTO expression. Reduced FTO subsequently caused reduced demethylation and instability of SMAD2 mRNA, which led to weaker anabolism and stronger catabolism in OA tissues than in normal tissues.

In summary, FTO plays an important role in many diseases; however, studies on its role in OA are limited. Our study contributes to our understanding of the relationship between FTO and OA and clarifies this relationship in detail using in vitro and in vivo data (Fig. 6). We also demonstrated that the mechanism of FTO in OA involves different tissues, including blood, thyroid, liver, bladder, and breast cancer. Furthermore, FTO and SMAD2 slow OA progression and are promising therapeutic targets for this disease.

## Supplementary information

Supplementary Information

## Data Availability

The data are available in a public, open access repository. All data relevant to the study are included in the article or uploaded as supplementary information.
