# Supplementary material for: FTO-mediated SMAD2 m6A modification protects cartilage against Osteoarthritis
Source: Exp Mol Med. 2024 Oct 3;56(10):2283–95. doi: 10.1038/s12276-024-01330-y (PMC11542000; doi:10.1038/s12276-024-01330-y)
Supplement: Supplementary file 1 — Supplementary Information [file 12276_2024_1330_MOESM1_ESM.pdf]

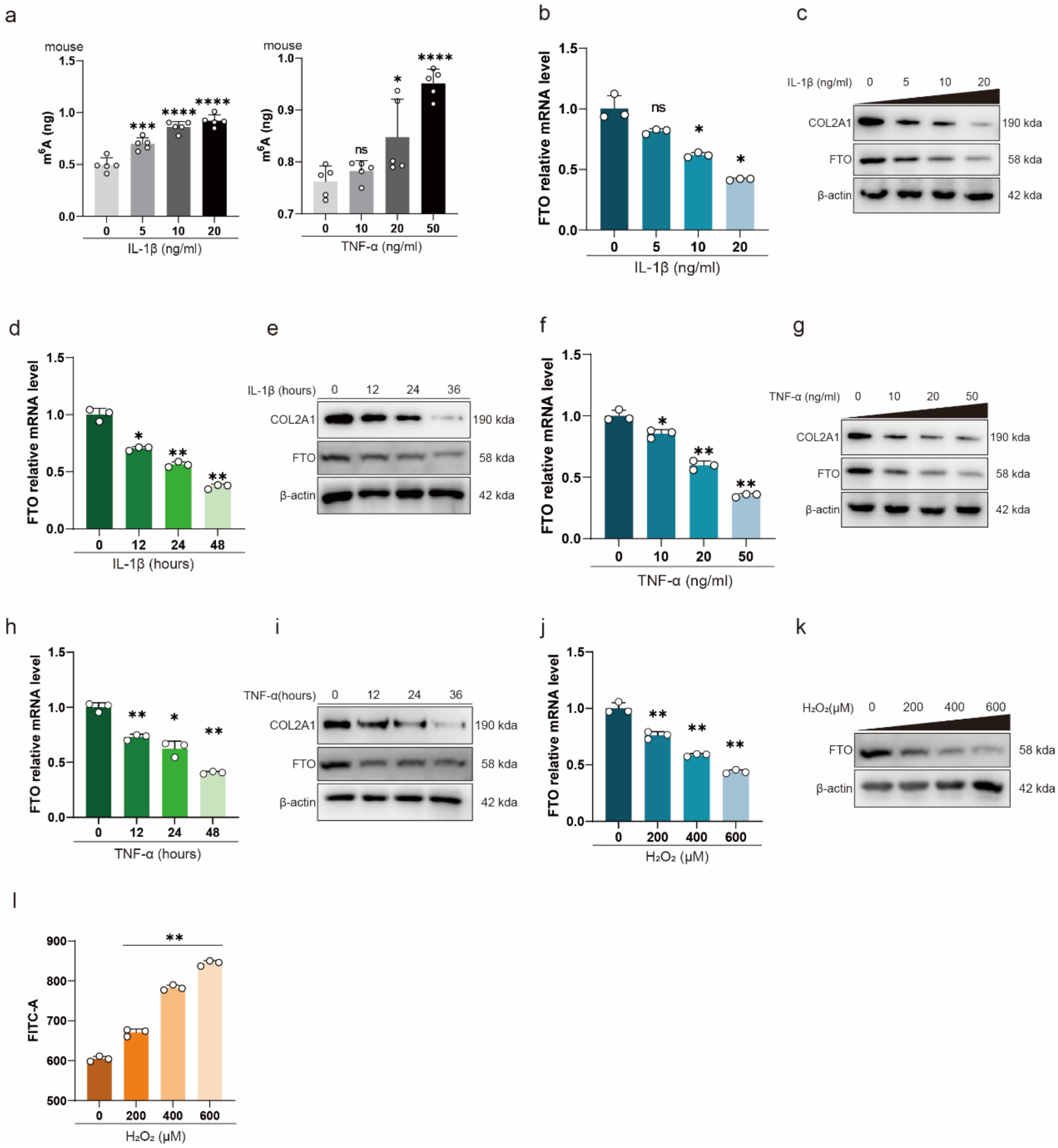

Supplementary Fig. 1 FTO expression decreased in Inflammatory environment and oxidative stress with the increase in m6A level. (a) m6A quantitative kit of mouse primary chondrocytes treated with IL-1β or TNF-α. (b) The mRNA expression level of FTO treated with IL-1β in a concentration-dependent manner in mouse chondrocytes. (c) Western blot detection of FTO expression treated with IL-1β in a concentration-dependent manner in mouse chondrocytes. (d) The mRNA expression level of FTO treated with IL-1β in a time-dependent manner in mouse chondrocytes. (e) Western blot detection of FTO expression treated with IL-1β in a

time-dependent manner in mouse chondrocytes. (f) The mRNA expression level of FTO treated with TNF-α in a concentration-dependent manner in mouse chondrocytes. (g) Western blot detection of FTO expression treated with TNF-α in a concentration-dependent manner in mouse chondrocytes. (h) The mRNA expression level of FTO treated with TNF-α in a time-dependent manner in mouse chondrocytes. (i) Western blot detection of FTO expression treated with TNF-α in a time-dependent manner in mouse chondrocytes. (j) The mRNA expression level of FTO treated with hydrogen peroxide in a concentration-dependent manner in mouse chondrocytes. (k) Western blot detection of FTO expression treated with hydrogen peroxide in a concentration-dependent manner in mouse chondrocytes. (l) Flow cytometry of mouse chondrocytes treated with hydrogen peroxide in a concentration-dependent manner; Data are representative of three independent experiments (c, e, g, i and k). *$p<0.05$, **$p<0.01$, ***$p<0.001$, ****$p<0.0001$, mean ± SD, one-way ANOVA.

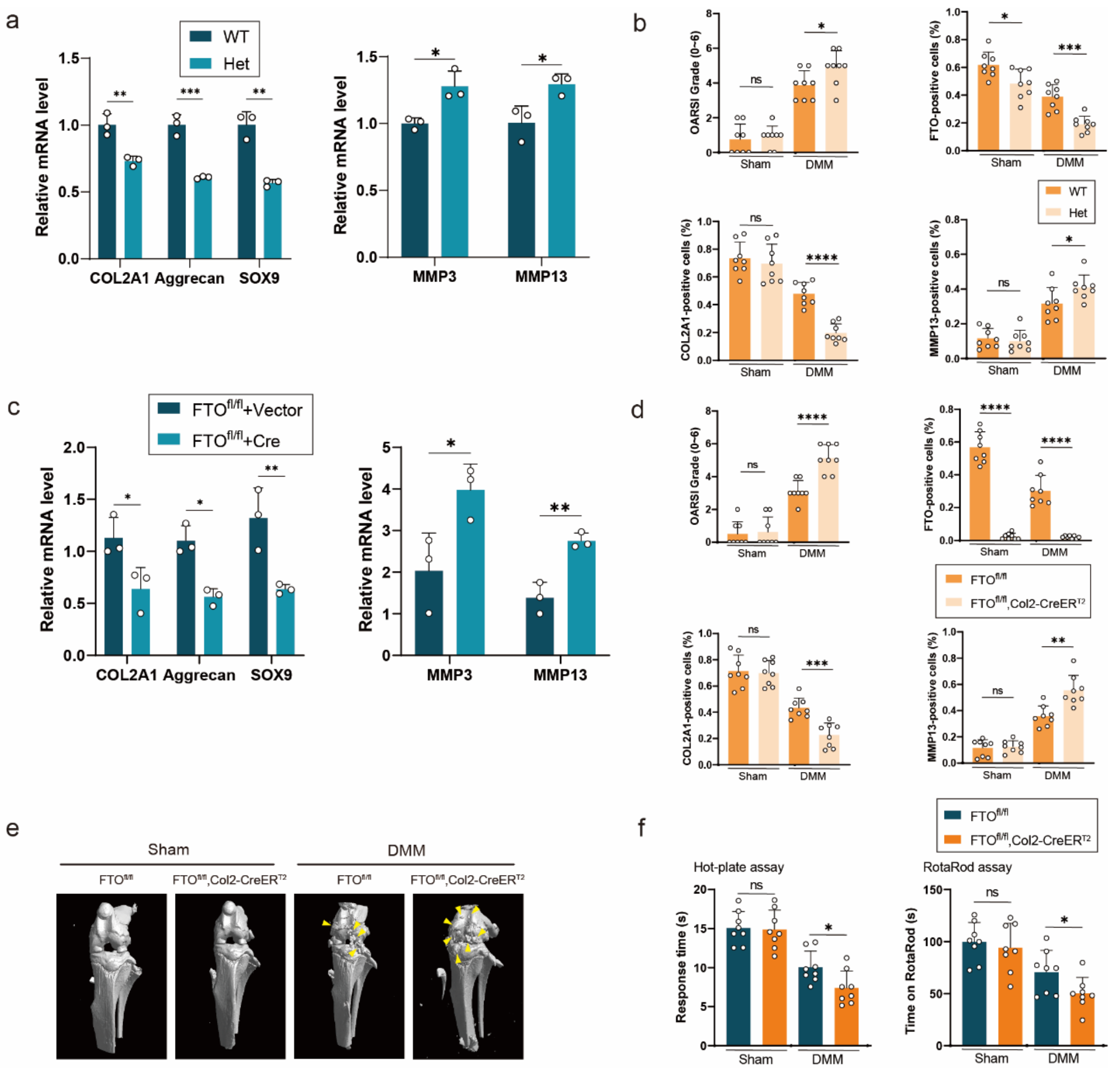

Supplementary Fig. 2 Reduced FTO contributed to the catabolic effects of chondrocytes *in vivo* and *in vitro*. (a) The mRNA expression level of COL2A1, Aggrecan, SOX9, MMP3 and MMP13 in primary chondrocytes isolated from $FTO^{+/+}$ or $FTO^{+/-}$ mice. (b) The Safranin O/Fast Green staining and immunohistochemistry (FTO, COL2A1 and MMP13) of knee joints of $FTO^{+/+}$ or $FTO^{+/-}$ mice that underwent DMM surgery or sham. n=8 per group. (c) The mRNA expression level of COL2A1, Aggrecan, SOX9, MMP3 and MMP13 in primary chondrocytes isolated from FTO-cKO mice cultured and transfected with Cre adenovirus or vector. (d) The Safranin O/Fast Green staining and immunohistochemistry (FTO, COL2A1 and MMP13) of knee joints of $FTO^{fl/fl}$

and FTO$^{fl/fl}$, Col2a1-CreER^{T2} mice that underwent DMM surgery or sham. n=8 per group. (e) The micro-CT images of knee joints of FTO$^{fl/fl}$ and FTO$^{fl/fl}$, Col2a1-CreER^{T2} mice that underwent DMM surgery or sham. Yellow arrows indicate the osteophytes. (f) The hot plate and rotarod of FTO$^{fl/fl}$ and FTO$^{fl/fl}$, Col2a1-CreER^{T2} mice that underwent DMM surgery or sham; *p<0.05, **p<0.01, ***p<0.001, ****p<0.0001, a, b, c, d and f mean ± SD, two-tailed t-test.

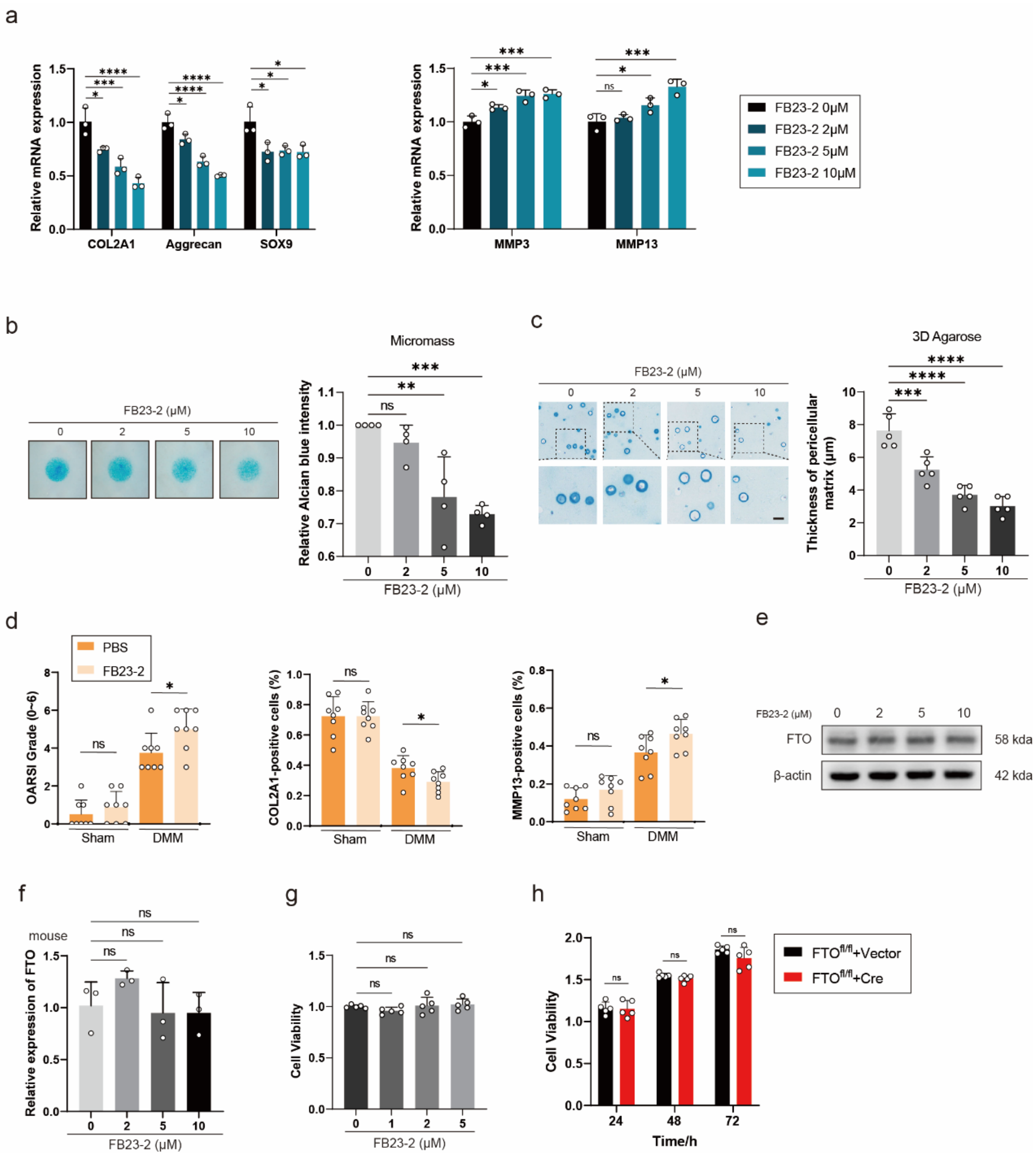

Supplementary Fig. 3 Effects of FTO inhibitors on anabolism and catabolism. (a) The mRNA expression level of COL2A1, Aggrecan, SOX9, MMP3 and MMP13 treated with FB23-2 in a concentration-dependent manner in mouse chondrocytes. (b) Chondrogenic matrix deposition (Alcian Blue staining) of FB23-2-treated mouse chondrocytes determined by micromass culture and quantified by ImageJ software. (c) 3D agarose culture of mouse chondrocytes (Alcian blue staining) showing the thickness of pericellular matrix. Scale bar, 20 μm. (d) The Safranin O/Fast Green staining and immunohistochemistry (COL2A1 and MMP13) of knee joints of mice that underwent DMM surgery or sham. Articular injection of FB23-2 or PBS was performed weekly. n=8 per group. (e) Western blot detection of FTO treated with FB23-2 in a concentration-dependent manner in mouse chondrocytes. (f) The mRNA expression level of FTO treated with FB23-2 in a concentration-dependent manner in mouse chondrocytes. (g) CCK8 detection of cell viability treated with FB23-2. (h) CCK8 detection of viability of primary cultured chondrocytes isolated from $FTO^{fl/fl}$ mice treated with Cre adenovirus or the vector; *p<0.05, **p<0.01, ***p<0.001, ****p<0.0001, D and F mean ± SD, two-tailed t-test; a, b, c and e mean ± SD, one-way ANOVA.

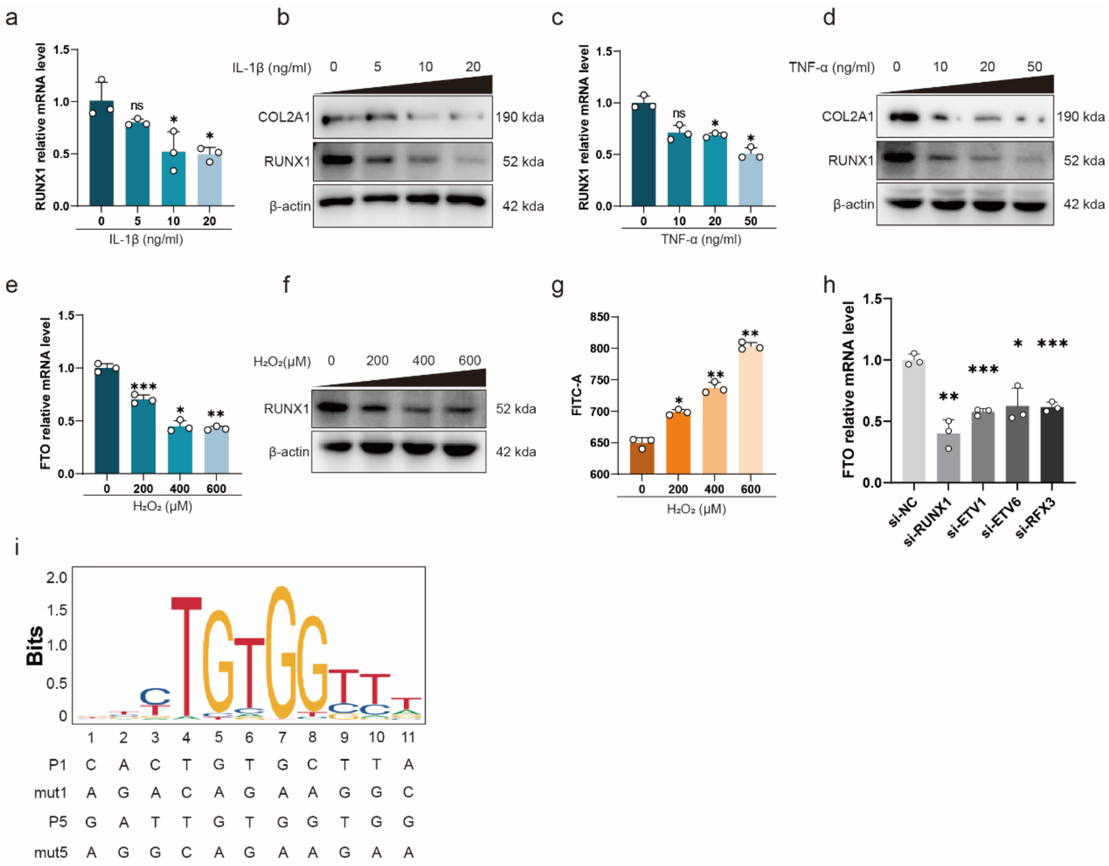

Supplementary Fig. 4 RUNX1 expression decreased in OA. (a) The mRNA expression level of RUNX1 treated with IL-1β in a concentration-dependent manner in mouse chondrocytes. (b) Western blot detection of RUNX1 expression treated with IL-1β in a concentration-dependent manner in mouse chondrocytes. (c) The mRNA expression level of RUNX1 treated with TNF-α in a concentration-dependent manner in mouse chondrocytes. (d) Western blot detection of RUNX1 expression treated with TNF-α in a concentration-dependent manner in mouse chondrocytes. (e) The mRNA expression level of RUNX1 treated with hydrogen peroxide in a concentration-dependent manner in mouse chondrocytes. (f) Western blot detection of RUNX1 expression treated with hydrogen peroxide in a concentration-dependent manner in mouse chondrocytes. (g) Flow cytometry of mouse chondrocytes treated with hydrogen peroxide in a concentration-dependent manner. (h) The mRNA expression level of FTO treated with siRNAs of

RUNX1, ETV1, ETV6 or RFX3 in mouse chondrocytes. (i) Mutant plasmids design at sites 1 and 5; Data are representative of three independent experiments (b, d and f). *$p<0.05$, **$p<0.01$, ***$p<0.001$, ****$p<0.0001$, H mean ± SD, two-tailed t-test; a, c, e and g mean ± SD, one-way ANOVA.

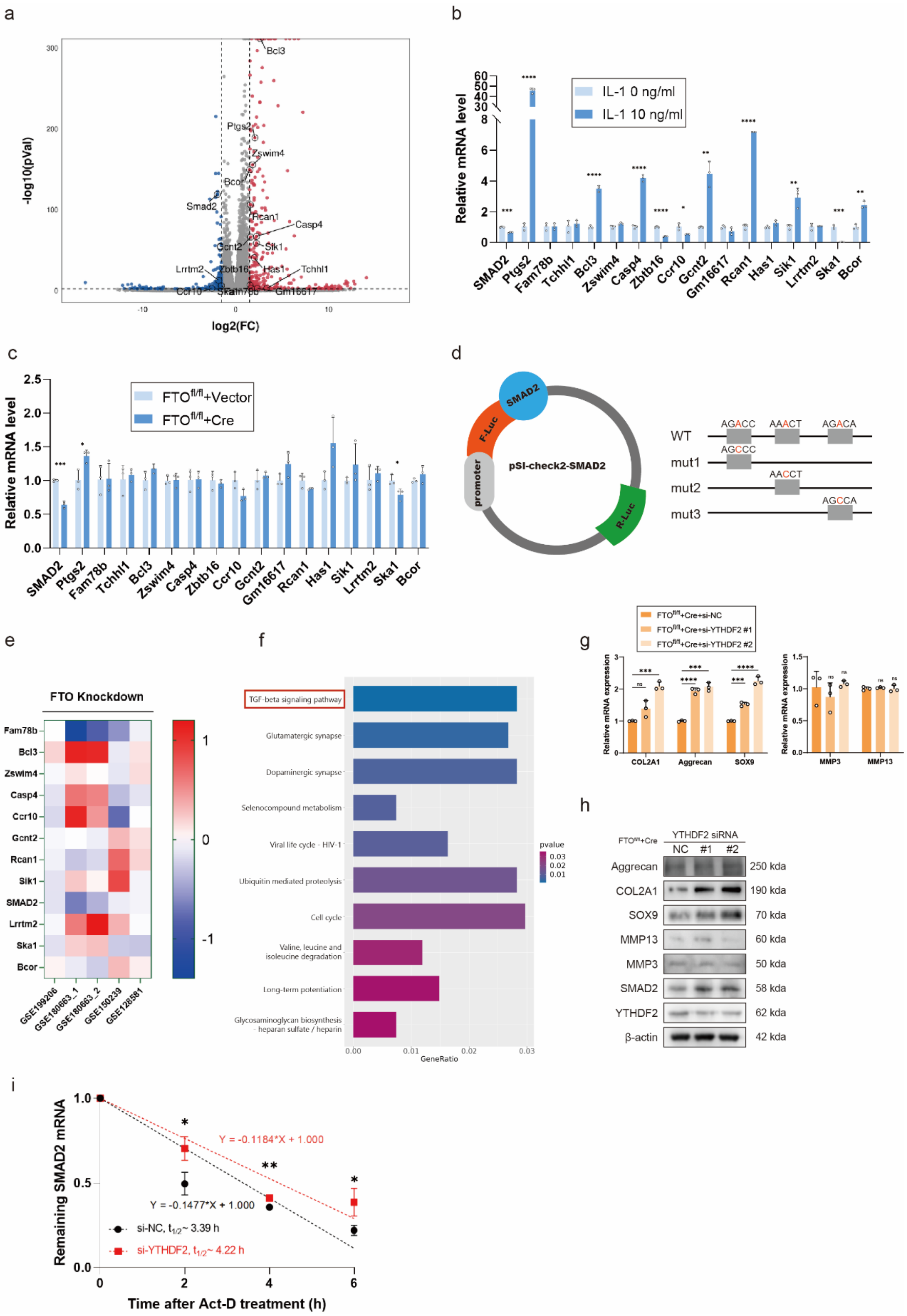

Supplementary Fig. 5 Regulation of FTO and YTHDF2 on SMAD2. (a) Volcano plot of RNA-seq of primary chondrocytes isolated from FTO-cKO mice cultured and transfected with Cre

adenovirus or vector. (b) The mRNA expression level of 17 target genes treated with IL-1β in mouse chondrocytes. (c) The mRNA expression level of 17 target genes in primary chondrocytes isolated from FTO-cKO mice cultured and transfected with Cre adenovirus or vector. (d) Mutant plasmids designed for luciferase assays. (e) Five databases on FTO knockdown. (f) KEGG analysis of GSE180663. (g) The mRNA expression level of COL2A1, Aggrecan, SOX9, MMP3 and MMP13 in primary chondrocytes isolated from FTO-cKO mice cultured and transfected with Cre adenovirus and siRNAs of YTHDF2. (h) Western blot detection of SMAD2, YTHDF2, COL2A1, Aggrecan, SOX9, MMP3 and MMP13 in primary chondrocytes isolated from FTO-cKO mice cultured and transfected with Cre adenovirus and siRNAs of YTHDF2. (i) The mRNA expression level of SMAD2 in mice chondrocytes treated with actinomycin D and si-YTHDF2 or si-NC at the indicated time points. Data are representative of three independent experiments. *p<0.05, **p<0.01, ***p<0.001, ****p<0.0001, mean ± SD, two-tailed t-test.

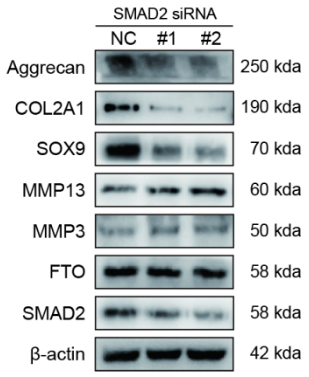

Supplementary Fig. 6 Western blot detection of SMAD2, FTO, COL2A1, Aggrecan, SOX9, MMP3 and MMP13 in primary mice chondrocytes treated with si-SMAD2 and si-NC. Data are representative of three independent experiments.

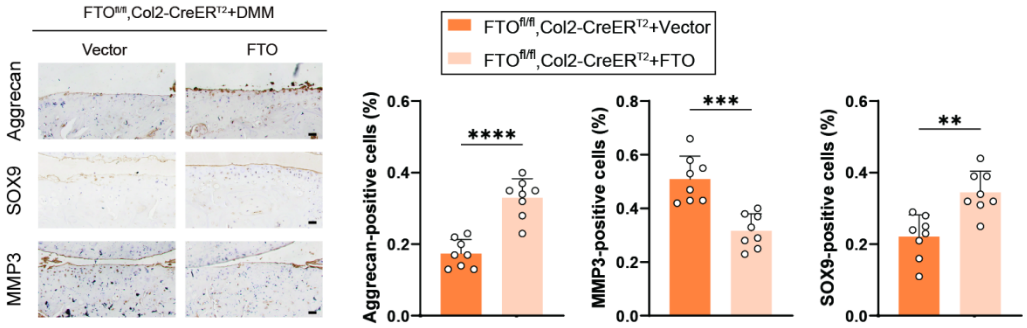

Supplementary Fig. 7 The immunohistochemistry (Aggrecan, SOX9 and MMP3) of knee joints of FTOfl/fl, Col2a1-CreERT2 mice that underwent DMM. Articular injection of FTO adenoassociated virus (AAV) or vector. n=8 per group. Scale bar, 20 μm. *p<0.05, **p<0.01, ***p<0.001, ****p<0.0001, mean ± SD, two-tailed t-test.
